# A wearable microwave instrument can detect and monitor traumatic abdominal injuries in a porcine model

**DOI:** 10.1038/s41598-021-02008-5

**Published:** 2021-12-01

**Authors:** Stefan Candefjord, Linh Nguyen, Ruben Buendia, Marianne Oropeza-Moe, Nina Gjerde Andersen, Andreas Fhager, Mikael Persson, Mikael Elam, Nils Petter Oveland

**Affiliations:** 1 Department of Electrical Engineering, Chalmers University of Technology, 412 96 Gothenburg, Sweden; 2 SAFER Vehicle and Traffic Safety Centre at Chalmers, Gothenburg, Sweden; 3 MedTech West, Sahlgrenska University Hospital, 413 45 Gothenburg, Sweden; 4 Department of Production Animal Clinical Sciences, Faculty of Veterinary Medicine, Norwegian University of Life Sciences, Sandnes, Norway; 5 Department of Anesthesiology and Intensive Care, Stavanger University Hospital, Stavanger, Norway; 6 Department of Clinical Neurophysiology, Sahlgrenska University Hospital, 413 45 Gothenburg, Sweden; 7 Department of Quality and Health Technology, Faculty of Health Sciences, University of Stavanger, Stavanger, Norway

**Keywords:** Diagnosis, Biomedical engineering

## Abstract

Abdominal injury is a frequent cause of death for trauma patients, and early recognition is essential to limit fatalities. There is a need for a wearable sensor system for prehospital settings that can detect and monitor bleeding in the abdomen (hemoperitoneum). This study evaluates the potential for microwave technology to fill that gap. A simple prototype of a wearable microwave sensor was constructed using eight antennas. A realistic porcine model of hemoperitoneum was developed using anesthetized pigs. Ten animals were measured at healthy state and at two sizes of bleeding. Statistical tests and a machine learning method were used to evaluate blood detection sensitivity. All subjects presented similar changes due to accumulation of blood, which dampened the microwave signal ($p < 0.05$). The machine learning analysis yielded an area under the receiver operating characteristic (ROC) curve (AUC) of 0.93, showing 100% sensitivity at 90% specificity. Large inter-individual variability of the healthy state signal complicated differentiation of bleedings from healthy state. A wearable microwave instrument has potential for accurate detection and monitoring of hemoperitoneum, with automated analysis making the instrument easy-to-use. Future hardware development is necessary to suppress measurement system variability and enable detection of smaller bleedings.

## Introduction

Traumatic injury is the leading cause of death for young people, and it shows a negative trend with increasing fatality rates^1^. Road traffic injury and falls are the most common causes of unintentional injury^1,2^. For patients with polytrauma, the main reason for death in the first 24 hours is often the combination of abdominal and pelvic injuries^3^. Early recognition of abdominal trauma requiring surgical intervention is essential to prevent death^3–5^. The problem is that injuries to hollow viscus, bleeding from solid organs or bone fractures from the pelvis may not be easily recognized. For every additional three minutes of delay for patients in need of emergency laparotomy due to hemoperitoneum (intra-abdominal bleeding), the probability of death increases 1%^5^.

Hemoperitoneum is the accumulation of blood in the cavity between the organs in the abdomen and the inner lining of the abdominal wall. The abdominal cavity can distend and is capable of holding more than five liters of blood for an average-sized individual, i.e. the entire volume of circulating blood. Massive intra-abdominal bleeding can lead to hemorrhagic shock and rapidly cause death if not treated adequately^6^. Blood transfusion is required as the first step if hemorrhagic shock occurs, and surgery or intervention radiology may be needed to locate and stop the source of bleeding, e.g. by clamping, embolization and ligating a burst vessel^4^.

Although CT is the gold standard to detect traumatic injuries including hemoperitoneum, this technique cannot be deployed widely in the prehospital setting due to its bulkiness and high cost, and it is unsuitable for field measurements at the scene of injury. The only commercially available diagnostic technique for prehospital detection of hemoperitoneum is ultrasonography^7^. In hypotensive abdominal trauma patients, a focused ultrasound protocol also known as the “Extended Focused Assessment with Sonography in Trauma” or eFAST exam, detects hemoperitoneum with high sensitivity and specificity^8^. A recent systematic review of nearly 25 000 patients showed pooled sensitivities and specificities for the detection of pneumothorax (69% and 99% respectively), pericardial effusion (91% and 94% respectively), and intra-abdominal free fluid (74% and 98% respectively)^9^. However, accurate hemoperitoneum detection using ultrasound requires a highly trained operator, and the method is therefore not currently in widespread prehospital use. The pooled sensitivity for ultrasound detection of intraperitoneal blood of 74% is also considered too low to be useful as a rule-out tool^9^. The patient’s clinical appearance after significant blood loss into the abdominal cavity may be subtle, without any changes in external dimensions of the abdomen and without obvious signs of peritoneal irritation. The advanced trauma life support (ATLS) curriculum highlights that any patient who has sustained injury to the torso from a direct blow, deceleration, blast or penetrating injury must be considered to have an abdominal visceral, vascular or pelvic injury until proven otherwise^4^. Hence, there is need for alternative methods for detection of hemoperitoneum not relying on clinical assumptions or operator-dependent image interpretations.

Whereas ultrasonography is the only commercially available method used for prehospital diagnosis of internal injuries, a few emerging technologies as operator independent alternatives are on the horizon. Electrical bioimpedance is a non-invasive, harmless, cost efficient, portable, rapid and easy to use technology; thus ideal for the prehospital environment. In Buendia et al.^10^ a diagnostic algorithm that accurately discriminated between patients suffering thoracic injuries and healthy subjects was designed using bioimpedance technology, and this technology warrants evaluation as a detector for abdominal bleeding. Other possible candidate technologies could be; (1) near-infrared spectroscopy, which is promising for prehospital cerebral bleeding detection^11,12^, but due to limited penetration depth may not be usable for hemoperitoneum detection; (2) micropower impulse radar technology, which however has shown limitations for effectively detecting pneumothorax in patients with blunt or penetrating chest trauma^13^.

Microwave technology has shown potential to be used in prehospital settings for internal lesion detection, including injuries in the torso region and traumatic brain injury^14–18^. The use of microwave technology can be motivated from the point of view of wave propagation in human tissues as it has an advantage over current state-of-the-art imaging or detection systems, thanks to the easy penetration of microwaves in the human tissues. It also has the advantage of lower impact of radiation (X-ray) and better robustness against shadowing. A microwave-based system is safe and without side effects.

Hitherto, the microwave technology has not been evaluated for detection of hemoperitoneum. In this study, we present the first prototype of a wearable microwave instrument that has the potential to detect and monitor bleeding in the abdomen, while being very easy-to-use by automated analysis. A wearable sensor would be advantageous for monitoring the progress of injury, which would be valuable for managing hemoperitoneum in the initial post-traumatic phases following injury. The system is intended to be worn during transportation in the ambulance, from the scene of an accident to the hospital. This typically means a period ranging up to a few hours. The emitted effect of the antennas is very low, $\sim 1$ mW, which is significantly lower than the maximum output effect of mobile phones. Therefore, we see no safety concerns for microwave radiation from usage of this wearable instrument.

For the first time, we evaluate microwave technology for assessment of hemoperitoneum, by using a realistic porcine model where bleedings of different sizes can be created in anesthetized pigs.

## Results

**Figure 1:**
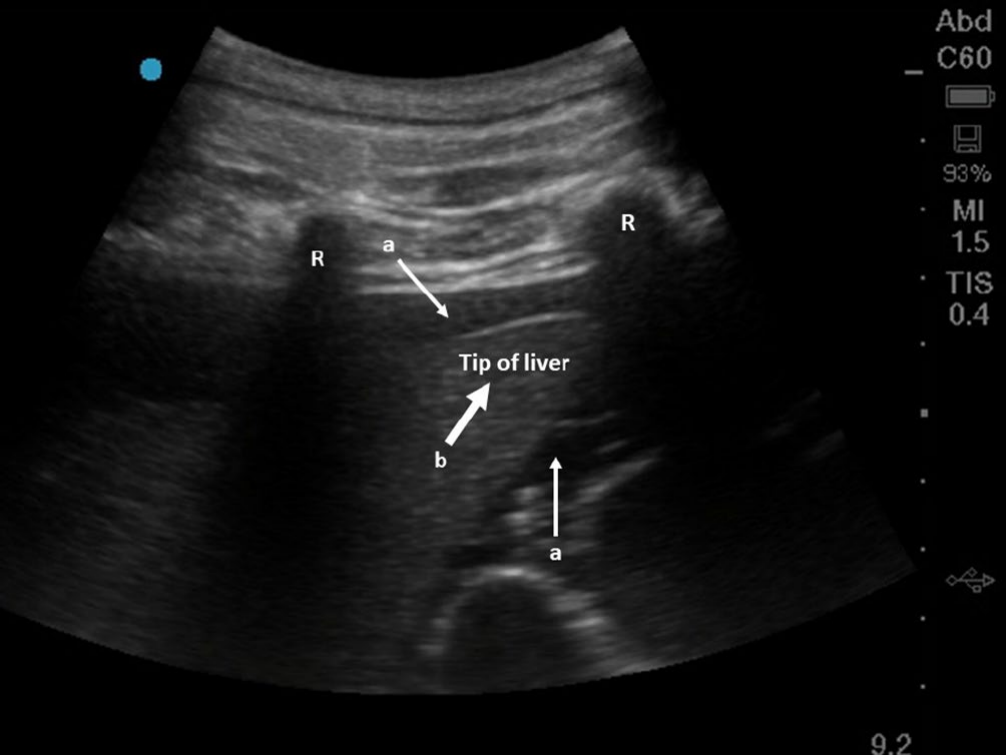
Ultrasound image showing hemoperitoneum in the right upper quadrant of a pig. The two white thin arrows (**a**) show accumulated blood around the liver. The thick white arrow (**b**) is the tip of the liver and the two ribs are marked with “R”.

**Figure 2:**
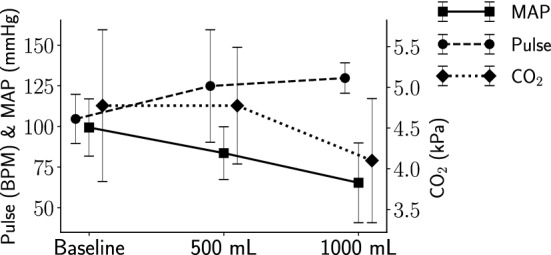
Vital signs at baseline, 500 mL and 1000 mL hemoperitoneum. The left y-axis indicates pulse rate and mean arterial pressure (MAP) levels and the right y-axis end tidal carbon dioxide ($\hbox {CO}_2$) levels. The whiskers show the standard deviations.

A total of ten measurements per class (i.e. baseline or hemoperitoneum) and pig were obtained from a total of ten pigs. An example of an abdominal bleeding visualized by ultrasound is shown in Fig. 1. Detailed animal information including weight, abdominal circumference and accumulated blood loss is presented in Table 1, and the vital sign trends in Fig. 2.

**Table 1:** Characteristics of study population. All the hybrid pigs ($n=10$) originated from purebred Duroc (DD) boars and crossbred Landrace Yorkshire (LY) sows, and were approximately four months old. The asterisk indicates a missing data point (measurement not recorded).

Animal	Bodyweight (kg)	Circumference abdomen (cm)
1	65	93
2	60	89
3	55	$*$
4	59	90
5	61	95
6	65	97
7	72	94
8	65	98
9	67	95
10	68	97

**Figure 3:**
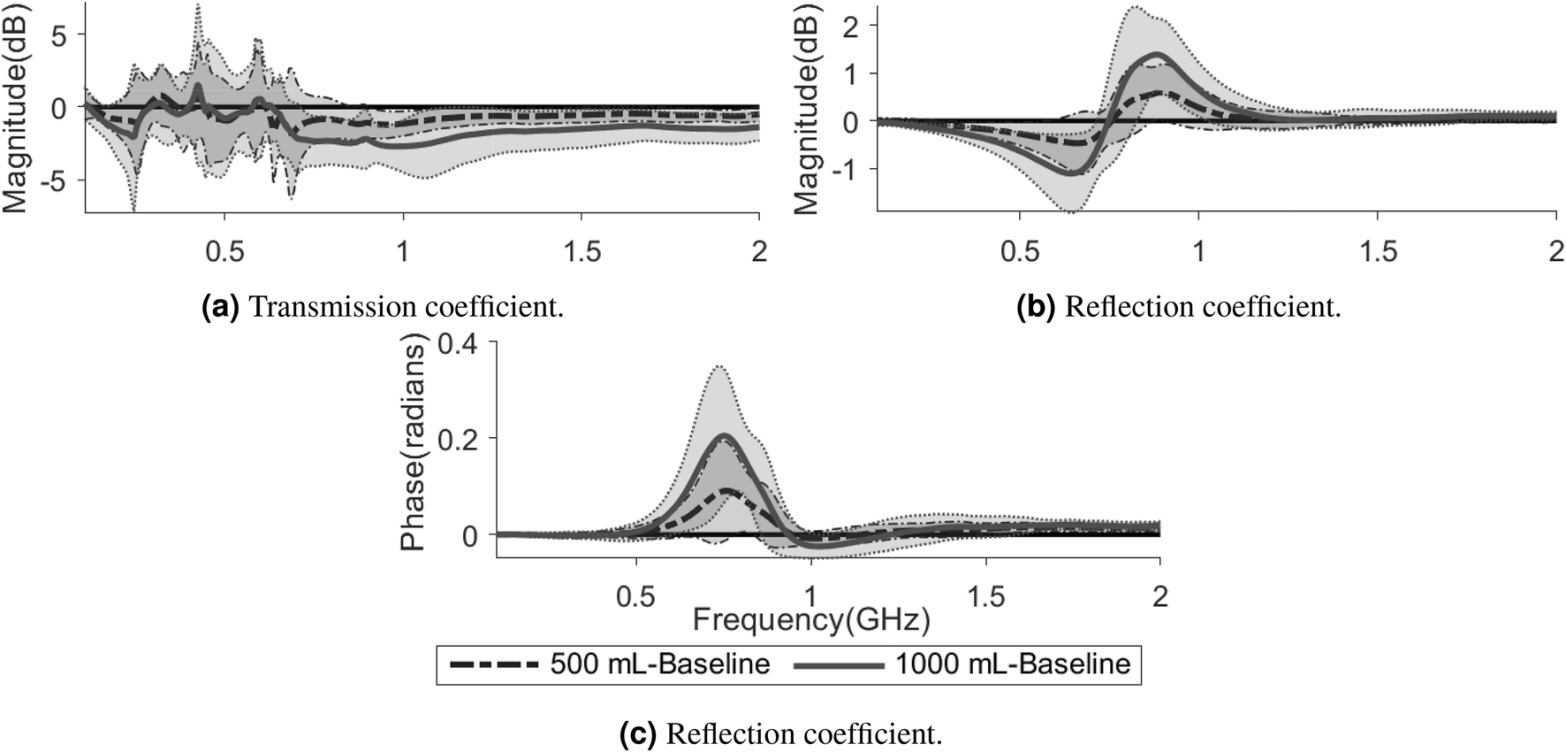
Some examples of the changes relative to baseline caused by hemoperitoneum in transmission coefficient magnitude (**a**) and reflection coefficient magnitude and phase (**b**, **c**). The lines show the average values across all subjects while the shaded areas show the standard deviations, with the darker shade for 500 mL and the lighter shade for 1000 mL. The solid lines at $y = 0$ are included to more clearly see the difference from baseline.

**Figure 4:**
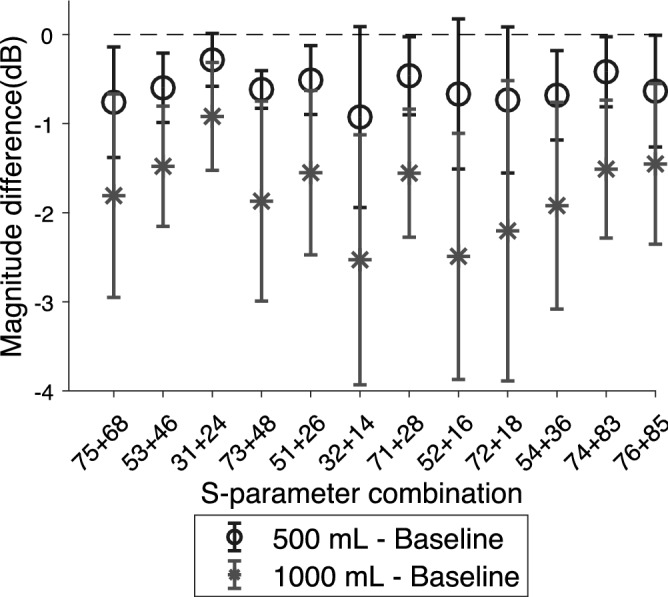
The average magnitude differences between baseline and 500 mL (circles) and 1000 mL (asterisks) hemoperitoneum, respectively, for all ten subjects and all symmetric pairs of transmission coefficients. The whiskers show the standard deviations.

In comparison with the baseline (healthy state) measurements, there are consistent changes due to hemoperitoneum among the tested subjects. For transmission coefficients, the signal magnitude is reduced as the volume of blood increases (Fig. 3a). The reflection coefficients show a decrease in magnitude below a certain frequency threshold close to the antenna resonance frequency, and an increase above that threshold, as compared to baseline (Fig. 3b). Meanwhile, the reflection coefficients indicate an increase in the phase of the signal (Fig. 3c). Figure 4 shows the changes in average magnitude for all symmetric pairs of transmission coefficients, as compared to baseline. The average magnitude drop in transmission coefficients when injecting 500 mL blood is around 0.3–1.0 dB, increasing to 1.0–2.5 dB for 1000 mL of blood (Fig. 4).

The Mann–Whitney U test shows statistically significant differences between hemoperitoneum and baseline for several S-parameters in both magnitude and phase (Table 2). Some transmission coefficients do not show statistically significant results for the test between baseline and the smallest bleeding (500 mL), while all reflection coefficients differ from baseline for both sizes of hemoperitoneum (Table 2).

**Figure 5:**
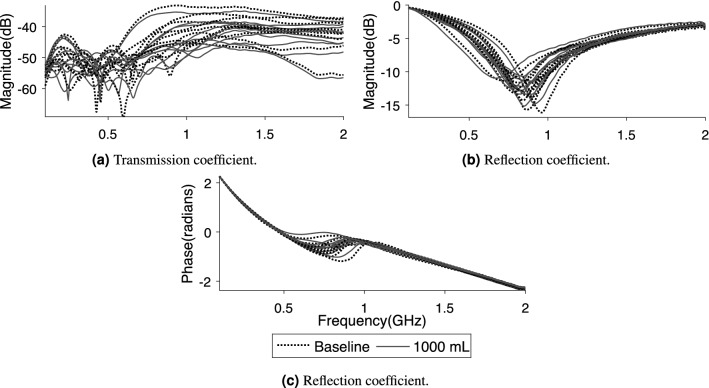
Examples ($S_{75+86}$ and $S_{77+88}$) of the large baseline difference between ten subjects in relation to the changes caused by hemoperitoneum. The data from the two groups completely overlap, and hemoperitoneum cannot be visually separated from healthy state.

Although the signal changes induced by hemoperitoneum are consistent among all subjects, there are large baseline differences between subjects that complicate differentiation (Fig. 5). The overall ($n = 10$ subjects) standard deviation in magnitude for all symmetric pairs of transmission coefficients is 4.0 dB, which is larger than drop in magnitude due to bleeding (Fig. 4).

**Table 2:**
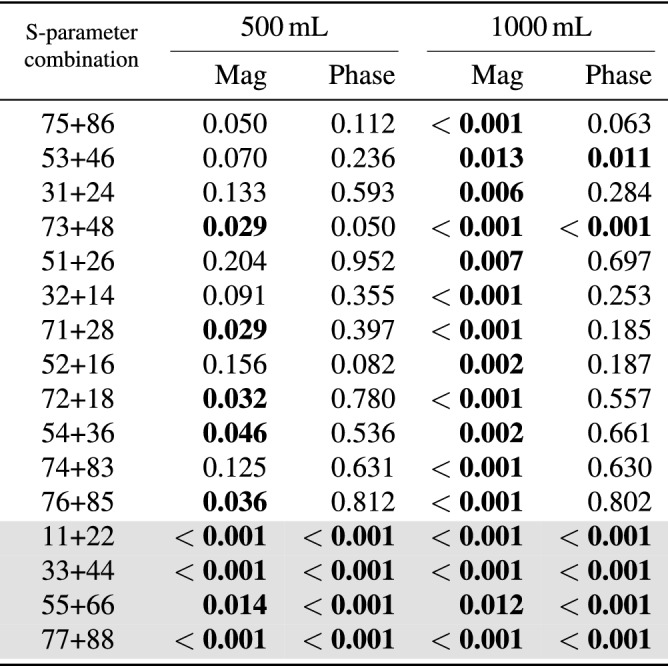
The outcomes of the Mann–Whitney U test when comparing baseline to hemoperitoneum for different S-parameter combinations. “Mag” and “Phase” represents the use of coefficient magnitude and phase, respectively. Statistically significant values ($p < 0.05$) are highlighted in bold text; unshaded rows are transmission coefficients whereas shaded rows are reflection coefficients.

**Figure 6:**
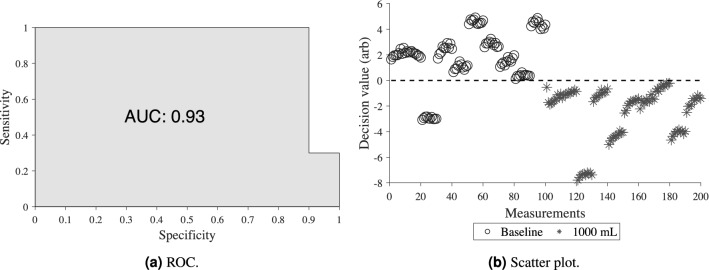
Receiver operating characteristic curve (ROC) (**a**) and scatter plot (**b**) of the best SVM classifier for differentiating 1000 mL hemoperitoneum from baseline.

The classification analysis shows the highest overall accuracy between the largest bleeding (1000 mL) and baseline using the sum of all reflection coefficients as input data, with 95% of observations having their class correctly predicted. Figure 6a shows the ROC curve with the AUC being 0.93. The specificity is 90% at 100% sensitivity. Figure 6b shows the scatter plot corresponding to the ROC curve.

## Discussion

In this study we have evaluated a prototype of a wearable microwave instrument for the task of detecting and monitoring hemoperitoneum, using a highly realistic porcine model. The results show that all subjects presented similar changes in microwave signal when blood accumulated in the abdominal cavity, which were statistically significant for all antenna combinations for the largest size of hemoperitoneum. The classification analysis yielded 95% accuracy for differentiating 1000 mL bleeding from baseline. Our findings also show that the large variation in signal baseline between individual animals complicates detection of hemoperitoneum.

### Observed changes in microwave signal and inter-individual variations

The presence of blood in the abdominal cavity clearly dampened the magnitude of microwave signals, and the dampening increased with a larger volume of blood. A dampening effect was expected due to the high conductivity of blood^17^. The statistical tests confirmed our observations that bleeding consistently dampened the microwave signal, showing low *p*-values for all S-parameter combinations for 1000 mL bleeding, whereas not all antenna combinations were statistically significant for 500 mL bleeding (Table 2). Furthermore, the reflection coefficients appeared to be especially sensitive to blood, since all S-parameter combinations were statistically significant for 500 mL and 1000 mL of blood, both for magnitude and phase (Table 2, shaded rows).

We observed large variations in baseline between the individual subjects (Fig. 5). These variations were larger than the change in magnitude caused by hemoperitoneum, and therefore complicated detection. These variations can stem from both anatomical variations between subjects and from measurement system variability. In our experience of microwave diagnostics nearby antennas will often exhibit a strong cross talk^17^. Since the antenna array geometry was not fixed in the current study, to allow flexibility to adapt the placement of antennas to suit animals of different sizes, the magnitude of cross talk is expected to change depending on the varying distances between antennas. We believe that the cross talk effect dominated over anatomical variability for the current experiments, but future studies are needed to establish the amount of variability stemming from different sources. We deem that the hardware design needs to be improved to suppress the variability due to cross talk, in order to succeed in developing a highly sensitive detection of hemoperitoneum.

### Classification

The classification analysis indicated that a wearable microwave instrument has potential to accurately detect hemoperitoneum (Fig. 6). The wearable microwave instrument had the highest classification accuracy and hence sensitivity to detect abdominal blood by using the reflection coefficient as input data. This is in agreement with the statistical tests that showed that the reflection mode is sensitive to presence of blood. The AUC was 0.93, which indicates that healthy subjects and patients with large abdominal bleedings can be effectively distinguished. Put differently, to detect all (i.e. 100% sensitivity) subjects with a 1000 mL abdominal bleeding, only one in ten healthy subjects will be incorrectly classified as false positive of having a bleeding (i.e. 90% specificity) (Fig. 6b). However, our goal is to develop an instrument that can detect hemoperitoneum with as high sensitivity as possible to avoid false negative results, that potentially could delay correct treatment and triage of polytrauma patients. The results using our prototype to detect blood in the abdominal cavity are promising and show the potential of microwave technology in medical diagnostic applications.

Ten subjects is a very small sample size for training a classifier^19^. Furthermore, the same individual was measured both in healthy state and in injured state, which differs from how a clinical study would be performed, where typically a patient would be measured in an injured state and comparisons would be made to a group of healthy volunteers^16^. Considering these factors, the classification analysis in the present study is merely an indication that the concept may be successful for future clinical use, and that further development and studies are warranted.

### Implications for future development of microwave device

The prototype instrument used in this study was very simple. In future development of an instrument that can be used for clinical measurements, it would be valuable to investigate different methods of suppressing cross talk between different antennas and/or decrease cross talk variability. Note that baseline variations will not affect the ability to monitor the size of a bleeding. As this study demonstrates an increase in the volume of blood produces prominent signal changes (Fig. 4), which is promising for monitoring a patient, e.g. during ambulance transport, to alert the prehospital staff of a massive internal blood loss.

Regarding antenna placement our findings indicate that antennas 7 and 8, which were placed close to the midaxillary line (Fig. 8), were among the most sensitive to the presence of blood (Table 2). This finding is also concordant with the ultrasound examinations, which showed that blood was accumulating predominantly in the vicinity of these antennas. Due to gravity and the dominant supine position in polytrauma patients, we therefore recommend placing antennas in proximity to the mid and posterior axillary line in future instrument designs.

## Conclusion

This pilot study on a realistic porcine model shows that a wearable microwave instrument has potential to accurately detect and monitor hemoperitoneum. Consistent changes and dampening of microwave signal were caused by accumulated abdominal blood. A challenge is to suppress measurement system variability with more effective instrument designs to decrease large baseline differences between individual subjects, to enable detection of smaller bleedings. Further system development and improved classifier algorithms are warranted, as is future larger scale animal experiments. When this is done clinical trials on polytrauma patients can commence.

## Methods

### Ethical considerations

This study is reported in accordance with ARRIVE guidelines. Ethical approval of experiments was granted by the Norwegian Food Safety Authority (approval no. 11933). All procedures were carried out at the Norwegian University of Life Sciences in Sandnes, Norway, and conformed to the Norwegian Animal Welfare Act (LOV-2009-06-19-97), the Regulation concerning the use of animals for scientific purposes (FOR-2015-06-18-761) and the Norwegian regulations on swine husbandry (FOR-2003-02-18-175).

### Animal model and anaesthesia

Healthy, prepubertal, female, approximately 16 weeks old hybrid finisher pigs (Duroc x Landrace/ Yorkshire, DDYL) of $63.7 \pm 5.0~\hbox {kg}$ body weight (mean ± standard deviation) were obtained from a commercial farm with high health status. The animals were vaccinated against porcine circovirus type 2 (PCV2) at three weeks of age. The Norwegian pig population is free of the suid herpesvirus 1 (SuHV-1), the African swine fever (ASF) virus, the Classical swine fever (CSF) virus, the transmissible gastroenteritis (TGE) virus, the porcine endemic diarrhea (PED) virus, Foot and Mouth disease (FMD) virus porcine reproductive and respiratory syndrome virus, Brucella suis and Mycoplasma hyopneumoniae. They were fasted one night (12 h) prior to the experiment with ad libitum access to water. They were sedated with an intramuscular injection of 1 mg/kg midazolam (Midazolam 5 mg/ml, B. Braun, Germany) and 15 mg/kg ketamin (Ketador vet. 100 mg/ml, Richter Pharma AG, Austria) in the morning of the experiment. After sedation, the animals were anaesthetized combining 300 microg/h fentanyl (Fentanyl 50 microg/mL, Hameln, Germany), 50 mg ketamine and 160 mg/h propofol (Propofol-Lipuro, 10 mg/mL B. Braun, Germany), intubated and positive pressure ventilated using a respirator set to a tidal volume of 400–600 mL, a respiratory rate of 16–18 breaths/min, to keep the initial end-tidal carbon dioxide level within the normal range (4.0 to 6.5 kPa). The inspiratory oxygen fraction was 30%. Maintenance of anaesthesia was achieved by the simultaneous intravenous infusion of 100–150 mg/h propofol and 300–600 microg/h fentanyl, and hydration with intravenous infusion of isotonic sodium chloride and Ringer acetate (in total 1000 mL per pig). All the animals were monitored by electrocardiography and their deep intranasal temperatures, invasive arterial blood pressures, oxygen saturations and end-tidal carbon dioxide levels were trended from recordings taken every fifth minute. The monitor used was Patient Monitor Agilent (V24C, M1205A, Agilent, Boeblingen, Germany).

### Porcine model of hemoperitoneum

The porcine model of pneumothorax developed by Oveland et al.^20–22^, was extended to include also the injuries hemoperitoneum and hemothorax. The thoracic injuries (i.e. pneumothorax and hemothorax) were modeled first and measured in a consecutive experimental series ending with hemoperitoneum. After the thoracic measurements were completed the pig was stabilized by relieving pressure from pneumothorax. The accumulated blood loss in the thoracic cavity was 750 mL. The additional modality bioimpedance was also used throughout the experimental series. The results from the thoracic microwave measurements and all bioimpedance measurements will be published elsewhere.

In total ten animals were measured, a sample size deemed sufficient for a first proof-of-concept study by indicating inter-individual variability and measurement system variability. No exclusion criteria were set, all animals and all measurements were used for data analysis. The study was open, not randomized, and potential confounders were not controlled. Each animal was measured both in healthy and injured state while keeping all other experimental factors as similar as possible, to isolate the effect of the injuries as far as possible. No separate control group was used, the measurements on healthy state were used as healthy control data, the rationale being to use fewer animals while still fulfilling the study aims.

The bristles on the animals’ chest and abdomen were removed using an electrical shaver and a three-way stopcock catheter (BD Connecta, 100 cm, BD Medical, USA) was inserted into the peritoneal space through a small laparotomy in the midline of the abdominal wall. The surgical incision was closed by subcutaneous and cutaneous stitches, with the catheter anchored to surrounding muscles and skin. Hemoperitoneum was induced by consecutive injections of blood into the abdominal cavity using a 50 mL syringe (Omnifix, 50 mL, B.Braun Medical, Germany), preceded by withdrawing blood from the femoral artery. Measurements were performed on two sets of blood volumes. The first volume of 500 mL of blood injected into the abdominal cavity was chosen as it is often referred to as a clinically significant blood loss and that the quantitative sensitivity of ultrasound in detecting free intraperitoneal fluid is poor under 400 mL^23^. Furthermore, we wanted to double that volume to 1000 mL of blood to be sure that we were well above the threshold where ultrasound can detect free abdominal fluid. At 1000 mL previous studies in humans have shown a 100% sensitivity of using ultrasound to detect fluid in the right upper quadrant (also known as Morison’s pouch)^23^.

The hemoperitoneum was confirmed by ultrasound examination using an M-Turbo ultrasound machine (SonoSite, USA). This was done by using a curvilinear abdominal ultrasound probe (M-Turbo C60, SonoSite, USA) scanning from the abdominal midline of the animal with the probe in the longitudinal direction and slovenly sliding the probe laterally out to the right upper abdominal quadrant of the animal and then to the left upper abdominal quadrant. The detection of accumulated free fluid in the abdominal cavity was then confirmed as black anechoic fluid as illustrated in Fig. 1.

### The wearable microwave instrument

**Figure 7:**
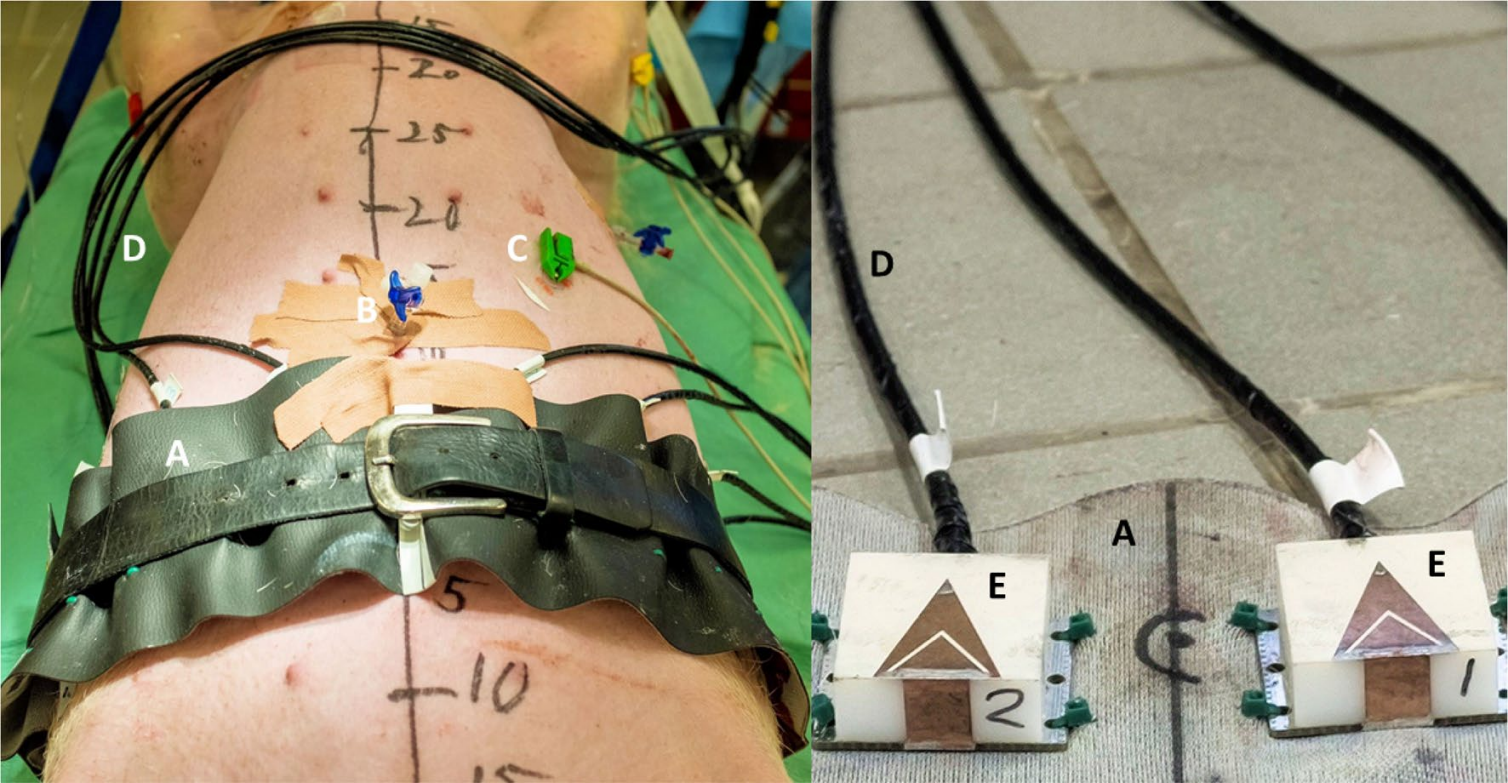
Experimental study setup. Left) Porcine model with a microwave belt (**A**) around the abdomen, the inserted catheter into the intraperitoneal cavity (**B**), ECG cable (**C**), and microwave cables (**D**). Right) Image of two of the microwave antennas (**E**) can be seen on the inside of the microwave belt (**A**).

**Figure 8:**
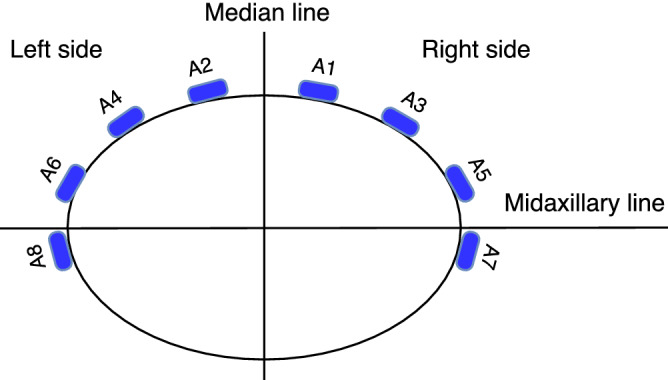
The geometrical arrangement of the eight antennas incorporated in the wearable microwave belt. The antennas were numbered so that even antennas were on the left side and odd antennas on the right side.

The experimental setup is shown in Fig. 7. The wearable microwave belt was constructed from an elongated piece of leather that could be wrapped around the body. It incorporated antennas described by Trefna and Persson^24^. Eight antennas were fitted onto the belt using cable ties, placed according to the pattern in Fig. 8. Each antenna has dimensions of length 37 mm, width 25 and thickness 14.4 mm, and constitute broad band patch antennas. The geometrical arrangement of the antennas was adapted for effective measurement of the abdomen. We refrained from placing antennas at the back of the subject, because such placement was deemed to be difficult to apply in a prehospital setting, where patients are generally placed in a supine position. The antennas were connected to a solid state switch matrix with eight ports, Ranatec RI2582 (Gothenburg, Sweden), which in turn was connected to a Keysight’s N7081A (Santa Rosa, CA, USA) microwave transceiver. The measurement procedure constitutes microwave transmission measurement between all possible combinations of antenna pairs. This procedure was automated and controlled via a PC. All hardware and software used for performing the measurements were provided by Medfield Diagnostics AB (Gothenburg, Sweden).

### Measurements

The microwave belt was fastened around the mid part of the pig’s abdomen as illustrated in Figs. 7 and 8, with the surface of the antennas in direct contact with the skin. An additional leather belt was strapped around the body and placed on top of the antennas (Fig. 7), to apply circular pressure to ensure good connectivity. After placement of the blood infusion catheter, but before injecting any blood into the abdominal cavity, baseline measurements representing a healthy state were performed. Then, hemoperitoneum of two sizes were created, 500 mL and 1000 mL. For both the healthy state and the two sizes of hemoperitoneum, ten consecutive microwave measurements were performed, which was important to capture data variability predominantly stemming from the pig’s respiratory movement.

### Microwave data

The raw collected microwave data are composed of scattering parameters (denoted S-parameters) of all possible antenna combinations. The S-parameters represent the relationship between the transmitted and received signal and are symbolized as $S_{ij}$, where *i* and *j* represent the transmitting antenna *i* and the receiving antenna *j*, respectively. $S_{ij}$ is also called reflection coefficient when $i=j$, i.e. the same antenna both transmits and receives, and transmission coefficient when $i \ne j$. With eight antennas, the total number of coefficients is 64 ($8 \times 8$), but due to the reciprocity of the transmitting and receiving antennas ($S_{ij} = S_{ji}$) only one of these symmetric coefficients is needed, resulting in 36 unique coefficients in total with 28 transmission coefficients and eight reflection coefficients.

The frequency range measured was 0.1–2 GHz at a step-size of 3.2 MHz, resulting in a vector with 630 measurement points for each coefficient. The chosen frequency range was the full range of the microwave transceiver, except that frequencies below 0.1 GHz were excluded because they exhibit poor signal to noise ratio.

### Data analysis

The presence of blood is expected to dampen the magnitude of the microwave signals and introduce patterns of changes in the scattering parameters that can be interpreted by a machine learning classifier^17^. The data analysis had two aims: (1) Verify that accumulation of blood dampens the signal magnitudes, and quantify other signal changes that were visualized from plotting and comparing data on bleeding versus baseline, using statistical tests; (2) Evaluate if a classifier can differentiate bleeding from baseline. MATLAB (version R2018a, MathWorks Inc., Natick, MA, USA) was used for all data analysis.

#### Preprocessing

The raw microwave data were preprocessed to transform and combine S-parameters to be suitable for data visualization and classification analysis. S-parameters are complex numbers ($a+bi$), and they were analyzed using magnitude (i.e. $\sqrt{a^2+b^2}$) and phase (i.e. $\arctan (b/a)$), separately, following common practice to analyze microwave data. For more effective visualization a smoothing algorithm developed by Eilers^25^, which is fast and give continuous control over smoothness, was applied to all S-parameters to diminish the effect of noise. Noise in the raw data was especially prominent for S-parameters with relatively long direct path between transmitting and receiving antenna, as expected due to the dampening from the tissue.

To compare baseline and hemoperitoneum, the magnitude and phase of symmetric coefficients between the left and right side of the median line (e.g. $S_{75}$ and $S_{86}$, Fig. 8) were summed (e.g. $|S_{75}|+|S_{86}|$ and $\angle S_{75}+\angle S_{86}$). The rationale was that the abdominal bleeding could spread into the whole abdominal cavity, but an even distribution of blood on the left and right side was not expected, due to anatomical asymmetries and small variations of the position for each animal (some degree of tilt towards one side would always be present). Thus, blood could accumulate predominantly in either left or right side, which also was confirmed by ultrasound examinations during the experiments. By summing signals from left and right sides, changes could be picked up regardless of the distribution of blood in the abdominal cavity.

#### Statistical tests

To assess whether differences between baseline and hemoperitoneum were statistically significant, the nonparametric Mann-Whitney U test was used, after recognizing that data were not normally distributed by means of the Anderson-Darling test. All requirements of the Mann–Whitney U test were fulfilled, with our assumption that the measurements were independent. Statistical significance was considered to be $p < 0.05$.

In the statistical tests, the magnitude and phase of each coefficient were tested separately. For each coefficient, data at all frequency steps were averaged, rendering a single value per coefficient. This procedure yielded 100 data points (10 pigs $\times$ 10 repeated measurements) per coefficient and class (i.e. baseline or hemoperitoneum class), which were used as basis for the tests.

#### Classification

Support vector machine (SVM) is a widely used machine learning technique for data classification that was used in this study for the task of differentiating hemoperitoneum from baseline measurements. The advantage of SVM is the ability to deal with high-dimensional data without a specific requirement on the minimum number of input samples^26^.

To avoid overfitting in training (a model being fitted to irrelevant information/noise), we utilized the leave-one-out (LOO) cross-validation scheme, which in each iteration removes one subject from the training set and saves it for testing. The diagnostic performance was evaluated using the classification accuracy (the percentage of correctly classified observations) and the area under the curve (AUC) calculated from the receiver operating characteristic curve (ROC)^27^.

The input data to the SVM classifier consisted of the whole frequency interval (0.1–2 GHz) for all S-parameters. In addition, some further tests were made by selecting a subset of different coefficient combinations, i.e. using only the reflection coefficients or only the transmission coefficients. Using the summation of all reflection/transmission coefficients was also analyzed. The rationale for these additional tests was due to the findings from plotting signals and the statistical tests that accumulation of blood was affecting S-parameters to different degrees and that the performance could potentially be improved by using a subset of data or derived features.

### Ethics approval

Ethical approval of experiments was granted by the Norwegian Food Safety Authority, and conformed to the Norwegian Animal Welfare Act, the Regulation concerning the use of animals for scientific purposes, and the Norwegian regulations on swine husbandry.

## References

[1] World Health Organization. *Injuries and Violence: The Facts* (2010).

[2] Tracy ET (2013). Pediatric injury patterns by year of age. J. Pediatr. Surg..

[3] Nast-Kolb D, Waydhas C, Kastl S, Duswald K-H, Schweiberer L (1993). The role of an abdominal injury in follow-up of polytrauma patients. Chirurg.

[4] American College of Surgeons. *Advanced Trauma Life Support (ATLS) Student Course Manual*, 10th edn. (2018).

[5] Clarke JR, Trooskin SZ, Doshi PJ, Greenwald L, Mode CJ (2002). Time to laparotomy for intra-abdominal bleeding from trauma does affect survival for delays up to 90 minutes. J. Trauma Inj. Infect. Crit. Care.

[6] Nunez TC, Cotton B (2009). Transfusion therapy in hemorrhagic shock. Curr. Opin. Crit. Care.

[7] Gillman LM, Ball CG, Panebianco N, Al-Kadi A, Kirkpatrick AW (2009). Clinician performed resuscitative ultrasonography for the initial evaluation and resuscitation of trauma. Scand. J. Trauma Resusc. Emerg. Med..

[8] van der Weide L (2019). Prehospital ultrasound in the management of trauma patients: Systematic review of the literature. Injury.

[9] Netherton S, Milenkovic V, Taylor M, Davis PJ (2019). Diagnostic accuracy of eFAST in the trauma patient: A systematic review and meta-analysis. CJEM.

[10] Buendia R (2017). Bioimpedance technology for detection of thoracic injury. Physiol. Meas..

[11] Peters J, Van Wageningen B, Hoogerwerf N, Tan E (2017). Near-infrared spectroscopy: A promising prehospital tool for management of traumatic brain injury. Prehosp. Disaster Med..

[12] Skrifvars MB, Aneman A (2020). How near is near infrared spectroscopy in pre-hospital care?. Acta Anaesthesiol. Scand..

[13] Rehfeldt M, Slagman A, Leidel BA, Möckel M, Lindner T (2018). Point-of-care diagnostic device for traumatic pneumothorax: low sensitivity of the unblinded pneumoscan$$^{\text{ TM }}$$. Emerg. Med. Int..

[14] Christopoulou M, Koulouridis S (2015). Inter-subject variability evaluation towards a robust microwave sensor for pneumothorax diagnosis. Prog. Electromagn. Res. M.

[15] Rezaeieh SA, Zamani A, Bialkowski KS, Abbosh AM (2017). Novel microwave torso scanner for thoracic fluid accumulation diagnosis and monitoring. Sci. Rep..

[16] Ljungqvist J (2017). Clinical evaluation of a microwave-based device for detection of traumatic intracranial hemorrhage. J. Neurotrauma.

[17] Candefjord S (2017). Microwave technology for detecting traumatic intracranial bleedings: Tests on phantom of subdural hematoma and numerical simulations. Med. Biol. Eng. Comput..

[18] Fhager A, Candefjord S, Elam M, Persson M (2018). Microwave diagnostics ahead: Saving time and the lives of Trauma and stroke patients. IEEE Microwave Mag..

[19] Fhager A, Candefjord S, Elam M, Persson M (2019). 3D simulations of intracerebral hemorrhage detection using broadband microwave technology. Sensors.

[20] Oveland NP, Sloth E, Andersen G, Lossius HM (2012). A porcine pneumothorax model for teaching ultrasound diagnostics. Acad. Emerg. Med..

[21] Oveland NP (2013). The intrapleural volume threshold for ultrasound detection of pneumothoraces: An experimental study on porcine models. Scand. J. Trauma Resusc. Emerg. Med..

[22] Oveland NP (2013). Using thoracic ultrasonography to accurately assess pneumothorax progression during positive pressure ventilation. CHEST.

[23] Branney SW (1995). Quantitative sensitivity of ultrasound in detecting free intraperitoneal fluid. J. Trauma.

[24] Trefna, H. & Persson, M. Antenna array design for brain monitoring. In *2008 IEEE Antennas and Propagation Society International Symposium*, 1–4 (Institute of Electrical and Electronics Engineers, San Diego, CA, 2008).

[25] Eilers PHC (2003). A perfect smoother. Anal. Chem..

[26] Liu L., W. X., Shen B. Research on kernel function of support vector machine. In *Advanced Technologies, Embedded and Multimedia for Human-Centric Computing* (eds. Huang, Y. M., Chao, H. C., Deng, D. J. & Park, J.) (2014).

[27] Hanley JA, Mcneil BJ (1982). The meaning and use of the area under a receiver operating characteristic (ROC) curve. Radiology.

